# Ivermectin treatment of *Loa loa* hyper-microfilaraemic baboons (*Papio anubis*): Assessment of microfilarial load reduction, haematological and biochemical parameters and histopathological changes following treatment

**DOI:** 10.1371/journal.pntd.0005576

**Published:** 2017-07-07

**Authors:** Samuel Wanji, Ebanga-Echi J. Eyong, Nicholas Tendongfor, Che J. Ngwa, Elive N. Esuka, Arnaud J. Kengne-Ouafo, Fabrice R. Datchoua-Poutcheu, Peter Enyong, Dalen Agnew, Rob R. Eversole, Adrian Hopkins, Charles D. Mackenzie

**Affiliations:** 1 Parasites and Vectors Research Unit, Department of Microbiology and Parasitology, Faculty of Science, University of Buea, Buea, South West Region, Cameroon; 2 Research Foundation for Tropical Diseases and Environment (REFOTDE), Buea, South West Region, Cameroon; 3 Department of Biological Sciences, Faculty of Science, The University of Bamenda, Bambili, North West Region, Cameroon; 4 Department of Zoology and Animal Physiology, Faculty of Science, University of Buea, Buea, South West Region, Cameroon; 5 Department of Pathobiology & Diagnostic Investigation, Michigan State University, East Lansing, MI, United States of America; 6 Department of Biological Sciences, Western Michigan University, Kalamazoo, Michigan, United States of America; 7 Mectizan Donation Programme, 325 Swanton Way, Decatur, Atlanta, Georgia, United States of America; 8 Filarial Programmes Support Unit, Liverpool School of Tropical Medicine, Liverpool, United Kingdom; McGill University, CANADA

## Abstract

**Background:**

Individuals with high intensity of *Loa loa* are at risk of developing serious adverse events (SAEs) post treatment with ivermectin. These SAEs have remained unclear and a programmatic impediment to the advancement of community directed treatment with ivermectin. The pathogenesis of these SAEs following ivermectin has never been investigated experimentally. The *Loa*/baboon (*Papio anubis*) model can be used to investigate the pathogenesis of *Loa*-associated encephalopathy following ivermectin treatment in humans.

**Methods:**

12 baboons with microfilarial loads > 8,000mf/mL of blood were randomised into four groups: Group 1 (control group receiving no drug), Group 2 receiving ivermectin (IVM) alone, Group 3 receiving ivermectin plus aspirin (IVM + ASA), and Group 4 receiving ivermectin plus prednisone (IVM + PSE). Blood samples collected before treatment and at Day 5, 7 or 10 post treatment, were analysed for parasitological, hematological and biochemical parameters using standard techniques. Clinical monitoring of animals for side effects took place every 6 hours post treatment until autopsy. At autopsy free fluids and a large number of standard organs were collected, examined and tissues fixed in 10% buffered formalin and processed for standard haematoxylin-eosin staining and specific immunocytochemical staining.

**Results:**

Mf counts dropped significantly (p<0.05) in all animals following ivermectin treatment with reductions as high as (89.9%) recorded; while no significant drop was observed in the control animals. Apart from haemoglobin (Hb) levels which recorded a significant (p = 0.028) drop post treatment, all other haematological and biochemical parameters did not show any significant changes (p>0.05). All animals became withdrawn 48 hours after IVM administration. All treated animals recorded clinical manifestations including rashes, itching, diarrhoea, conjunctival haemorrhages, lymph node enlargement, pinkish ears, swollen face and restlessness; one animal died 5 hours after IVM administration. Macroscopic changes in post-mortem tissues observed comprised haemorrhages in the brain, lungs, heart, which seen in all groups given ivermectin but not in the untreated animals. Microscopically, the major cellular changes seen, which were present in all the ivermectin treated animals included microfilariae in varying degrees of degeneration in small vessels. These were frequently associated with fibrin deposition, endothelial changes including damage to the integrity of the blood vessel and the presence of extravascular erythrocytes (haemorrhages). There was an increased presence of eosinophils and other chronic inflammatory types in certain tissues and organs, often in large numbers and associated with microfilarial destruction. Highly vascularized organs like the brain, heart, lungs and kidneys were observed to have more microfilariae in tissue sections. The number of mf seen in the brain and kidneys of animals administered IVM alone tripled that of control animals. Co-administration of IVM + PSE caused a greater increase in mf in the brain and kidneys while the reverse was noticed with the co-administration of IVM + ASA.

**Conclusions:**

The treatment of *Loa* hyper-microfilaraemic individuals with ivermectin produces a clinical spectrum that parallels that seen in *Loa* hyper-microfilaraemic humans treated with ivermectin. The utilization of this experimental model can contribute to the improved management of the adverse responses in humans.

## Author summary

The pathogenesis of the serious adverse reactions that occur in patients carrying high loads (usually > 30,000mf/mL) of circulating *Loa loa* parasites and treated with ivermectin has to date not been clearly defined at the tissue level. These reactions, which can result in the death of many of those affects and cause permanent mental damage in others, inflicts considerable disruption to the important mass treatment programs to control and eliminate filariae where ivermectin is used. The recently described baboon model of hyper-microfilariaemia has been used here to study the pathological changes occurring after ivermectin treatment; the clinico-pathological presentation following treatment in baboons appears to be closely related to those seen in humans. The tissue changes seen suggest that the primary pathological event following ivermectin treatment is a vasculopathy associated with intra-vascular microfilarial destruction within smaller blood vessels that damages them and leads to changes in the tissues supplied by these vessels. The effects of cortisone and aspirin on these changes are also addressed in this study.

## Introduction

*Loa loa* is a parasitic filarial nematode of humans, restricted to the rainforest and forest fringes of West and Central Africa [1], causing the relatively well-tolerated disease-loiasis. Loiasis has two very characteristic clinical features: Calabar swellings, corresponding to an angioedema of allergic nature [2] and the passage of an adult worm under the conjunctiva (eyeworm). Other complications, though rare, include nephropathy [3], cardiomyopathy [4], retinopathy [5], arthritis [6], lymphangitis [7], peripheral neuropathy [8] and encephalopathy [9].

Loiasis is an infection of public health importance, essentially not because of its own clinical manifestations, but because it is associated with serious adverse events (SAEs) following the administration of ivermectin. These SAEs have a negative impact on the ongoing control of onchocerciasis and lymphatic filariasis with ivermectin distribution in areas of co-endemicity with *Loa*. The occurrence of *Loa-*associated SAEs with programs for these two major filariae (onchocerciasis and lymphatic filariasis) in *L*. *loa* endemic areas has been increasingly reported over the past decade. Clinical problems seen include severely disabling and potentially fatal, encephalopathy as well as other permanent clinical changes. Current observations have indicated that the risk of developing marked or serious reactions is significantly higher when the *L*. *loa* loads exceeds 8,000mf/mL; the severity of these reactions becomes more common in patients with the risk of these happening and being severe much higher in patients with > 30,000 mf/mL, with the risk of problems being very high with loads above with > 50,000mf/mL [10–13].

Although the epidemiological mapping and clinical description of these Loa-associated SAEs is now well known, the pathogenesis and the optimal approaches to treatment still remain obscure. An increase in the presence of *L*. *loa* in the brain tissue has been proposed as an important feature of the condition, and a central role for vascular pathology in the adverse reaction also proposed [14, 15]. Understanding the mechanism of pathogenesis of the post-ivermectin events in these heavily infected individuals is an important step towards the development of better treatments and responses to this unfortunate pathology. The lack of data from human cases and especially the lack of autopsy material have hindered the progress in the treatment and prevention of this condition. The previous lack of a relevant experimental model also contributed to the lack of solid information on these adverse reactions and delay in developing suitable clinical treatment protocols. A better understanding of the pathogenesis of this post-treatment condition is urgently needed.

After several consultation meetings held by the Scientific working Group on SAE in *L*. *loa* endemic countries, [16], it was strongly recommended that a non-human primate (NHP) animal model of *Loa* encephalopathy be developed. Non-human primates (NHP) are therefore considered the best models for the much needed investigation in human loiasis, especially as suitable *in vitro* models for loiasis have not yet been developed, and indeed such artificial models are not easily extrapolated to humans [17–19]. Although the drill is an excellent experimental host, there are ethical concerns with using this protected animal for research, and it is no longer used in biomedicine. The use of *Patas* monkeys also is limited as the parasite behaves differently from the same way that it does in the more human-like drill [20].

As recently described, the baboon (*P*. *anubis*) is a potentially useful model for studying the mechanisms behind the SAEs that develop in *L*. *loa* infected people as the parasite in this animal behaves essentially in the same way as it does in the drill. The use of baboons in biomedical research is accepted by the International Union for Conservation of Nature (IUCN) [21] and so Wanji *et al* developed a hyper-microfilaraemic *Loa*/baboon (*Papio anubis*) experimental model and characterized it parasitologically, haematologically and biochemically [22]. In this model microfilariaemia increases steadily in all infected animals and reaches a peak at 18 months post infection (MPI); by 10 MPI >70% of animals have mf > 8,000 mf/mL, at 18 MPI >70% of animals have mf >30,000mf/mL and 50% of animals have mf >50,000mf/mL. In this study, the three most significant alterations seen were: increased eosinophil, creatinine and gamma-GT levels. An intriguing question that now emerges is whether these specific alterations bear any role in the development of the post ivermectin SAEs. It also remains to be demonstrated whether if these *Loa* hyper-microfilaraemic baboons are treated with ivermectin-IVM (i) There will be a drastic drop in microfilariae load? (ii) Treatment will lead to serious clinical manifestations? (iii) There will be changes in blood chemistry and haematological parameters? (iv) Microfilariae will be seen in the different tissues and their distribution? (v) Which lesions will be seen and what are their characteristics (vi) and if the co-administration of ivermectin with either aspirin or prednisone will affect points i-v above. Therefore, this present study was carried out to treat *Loa* hyper-microfilaraemic animals such as to validate the model as a surrogate for the human condition, and to provide answers to the questions above.

## Materials and methods

### Animals

The acquisition, care and ethical concerns on the use of these animals have already been described in the preceding paper [22]. The animals for this study were gotten from the 15 baboons that had been characterised parasitologically, biochemically and haematologically in the preceding paper [22].

### Eligibility criteria and randomization into trial groups

**Fig 1:**
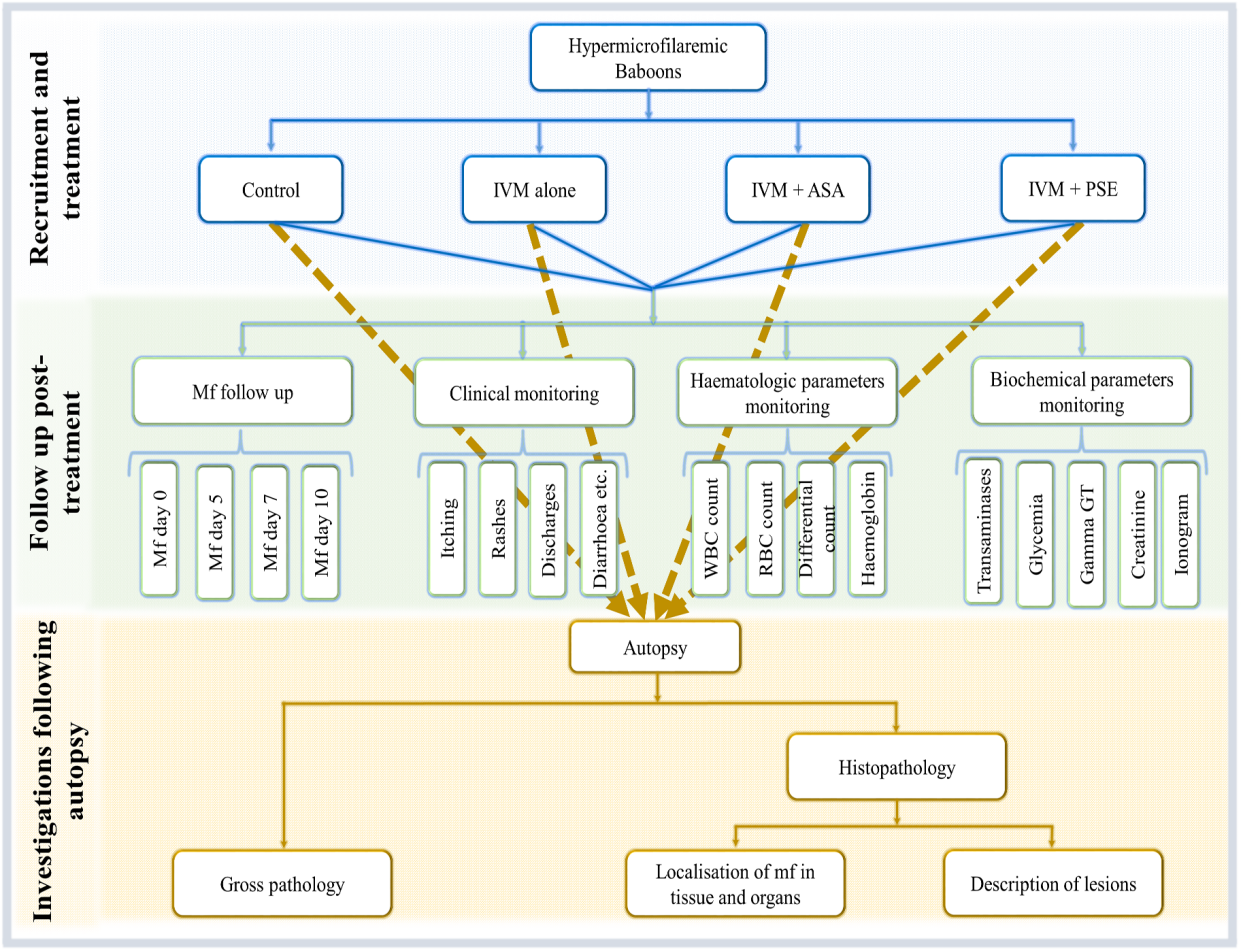
The study design.

Baboons to be included in the trials were chosen amongst the experimentally infected 15 animals. The inclusion criterion for animals to enter the drug treatment phase was a microscopically confirmed microfilariaemia of >8,000mf/mL whilst the exclusion criterion was a microfilariaemia of <8,000mf/mL. Ultimately, 12 infected splenectomised baboons were included in this phase of the study. The 12 baboons were randomised into 4 arms, with each treated group consisting of three animals. Eligible baboons were randomly assigned to the different treatment arms using a computer generated randomisation list. The randomisation was performed by a statistician not otherwise engaged in the study. The initial treatment allocation and the number of days animals were to be monitored were concealed but the subsequent administration of medication(s) was open label. Animals were placed into three different experiments based on the number of days animals were to be monitored; animals in experiment 1 were monitored for 5 days, those in experiment 2 for 7 days and those in experiment 3 for 10 days. After the randomization into treatment groups, the study was designed according to the flow diagram shown in Fig 1.

### Collection of pre-treatment and post-treatment data

Ten mL venous blood samples were collected under anesthesia (0.1 mL ketamine) and aseptic conditions from the femoral vein of each animal for parasitological (*L*. *loa*), haematological and biochemical analyses on Day 0 before treatment and on Days 5, 7 or 10 post treatment depending on the animals experimental group by a licensed veterinarian. Calibrated thick blood smears were prepared by spreading a 50 μL venous blood sample from a 75 μL non-heparinised capillary tube, onto a clean slide over an area of 1.5 cm x 2.5 cm for both the pre-treatment and post treatment samples.

The haematological parameters assessed were haemoglobin (Hb), red blood cell count (RBC), total white blood cell count (WBC) and white cell differential count in which neutrophils, eosinophils, basophils, monocytes and lymphocyte counts were measured. These analyses were performed on the pre- and post-treatment samples. The biochemical parameters quantified were grouped into the liver enzymes consisting of serum glutamate-pyruvate transaminase (SGPT), serum glutamate-oxaloacetate transaminase (SGOT) and serum γ-glutamyl transferase (γ-GT); blood biochemistry compounds comprising of creatinine and glucose; and blood elemental biochemistry comprising of calcium and potassium. The biochemical parameters were quantified using spectrophotometric kits obtained from HUMAN (www.human.de, Germany) as per the manufacturer’s instructions. The parasitological, haematological and biochemical analyses were performed on the pre- and post-treatment samples as described previously in Wanji *et al*., [22].

### Administration of drugs

The ivermectin used for this study was provided by the Mectizan Donation Program (MDP), and the aspirin (ASA) and prednisone (PSE) used in the study acquired commercially from Bayer and Ranbaxy laboratories Ltd., respectively. The animals were administered drugs via the oral route by a licensed veterinarian. Group 1 animal served as controls and received no drugs. Group 2 animals (IVM) received ivermectin alone. Group 3 animals (IVM+ASA) received IVM and 3 days after IVM administration, they received aspirin (1 tablet of 500mg 2 times a day) for two days; Group 4 animals (IVM+PSE) received IVM and 3 days after IVM administration, they received PSE (4 tablets of 5mg 2 times a day) for two days. The standard doses of ivermectin (150μg/kg of body weight), 500mg of ASA and 5mg of PSE were used for this study.

### Clinical monitoring

Clinical surveillance was carried out for up to 10 days. Following treatment, animals were closely monitored every day on a six hourly basis. Animals were assessed for general agility, meningeal signs, discharges, papillary reflex, diarrhea, weakness, conjunctival haemorrhage, dyspnea, itching (excoriations), muscle aches, restlessness, gland pain and gland tenderness, presence of rashes, pinkish ears, joint pain and swollen face. The agility and playing habits of the monkeys were assessed by the animal caretaker who was familiar with the normal behaviour of these baboons. Rashes were considered absent when none were found on the body of the animal, mild when < 3%, moderate when 3–10%, severe when 11–50% and unbearable when > 50%, of the body surface was affected. Itching was considered absent when no excoriations were found on the body of the animal, to be mild when < 3% of the body surface had excoriations, moderate when 3–10%, severe when 11–50%, and unbearable when > 50% of the body surface had excoriations. Gland pain/tenderness was assessed by palpating collections of lymph nodes in the neck, axillae and inguinal regions, and were considered as absent if no enlarged nodes were found; mild when the enlarged nodes were localized i.e. 1–2 enlarged nodes were found in one body area; moderate if 3–5 enlarged nodes were found in one body area; severe when 3–7 enlarged lymph nodes were generalized i.e. found in 2 or more body areas and unbearable when > 10 enlarged lymph nodes were generalized. Restlessness was assessed by counting the number of times the animals could not sit still; restlessness was considered absent when the animals sat still all the time, mild when it could not be still for less than 5 times, moderate when it could not be still for about 8–15 times, severe when it could not be still for 15 times and unbearable when the animal could not remain sitting still. Vital signs like rectal temperature, pulse rate (PR) and respiratory rate (RR) were also recorded. Animals in Experiment 1 were monitored every 6 hours for 5 days for a period of 120 mins, those in Experiment 2 every 6 hours for 7 days for a period of 168 mins while those in Experiment 3 were monitored every 6 hours for 10 days for 240 mins before autopsy.

### Pathology

The animals were euthanized by the administration of 10 mL of 5mg ketamine (Imalgene, Merial, France) and autopsies performed immediately. Gross organ and pathophysiologic abnormalities were observed during necropsy. Organs observed were the skin, brain, liver, lungs, diaphragm, pancreas, kidneys, heart, digestive tract and lymph nodes. These organs were collected and fixed in 10% formol (3% formaldehyde). Following paraffin embedding and sectioning (5 μm) the tissues were stained with a range of histochemical (haematoxylin-eosin) and immunochemical reagents (REF-350 was used to identify thrombi and UCH-ICC was used to identify the presence of macrophage cell lines) using standard immunocytochemical procedures from the Department of Pathology/Histology of the Michigan State University, Michigan, U.S.A.

### Microscopic examination of stained tissue sections

Slides with stained tissues of the brain, liver, lungs, skin, diaphragm, pancreas, kidneys, heart, digestive tract and lymph nodes were used to estimate *Loa* mf numbers and also analysed for pathologic changes. Ruled out papers of 50mm2 and 12.5mm2 were used to carve out areas on the prepared slides for organs with large surface areas (e. g. brain, kidney, lungs, etc.) and those with small surface areas (e. g. skin, lymph nodes, pancreas, etc.), respectively. The slides were rapidly focused at x10 to trace out the borders of the marked area and to check for Loa mf, after which the x40 objective was used to confirm the presence of *Loa* parasites and estimate the *Loa* mf numbers. After the counts had been made, the distribution of mf/25mm^2^ in all organs was calculated. The histopathological tissues were observed and interpreted by two trained histopathologists (CDM, DA).

### Data analysis

Normal ranges for various vital signs (temperature, respiratory rate (RR) and pulse rate (PR) in baboons were gotten from the Association of Primate Veterinarians Primate Formulary (1999) for baboons housed individually in large custom cages. The data were entered into Epi-Info version 3.5.3 (C.D.C. Atlanta, GA, U.S.A.) and analysed using the Software Package SPSS version 20. Graphs were drawn using the Graph Pad Prism version 5 for windows, Graphpad Software, San Diego California, U.S.A. Descriptive statistical analyses were performed to compute the mean, median and standard deviations of Loa microfilarial counts, haematological and biochemical parameters in the animals pre- and post-treatment. The percentage reduction of peripheral mf following treatment in the different baboons was calculated using the formula below:

$$\%\;\mathrm{reduction}\;\mathrm{of}\;\mathrm{mf}\;=\frac{\mathrm{mf}\;\mathrm{before}\;\mathrm{treatment}\;\text{-}\;\mathrm{mf}\;\mathrm{before}\;\mathrm{treatment}\;x\;100}{\mathrm{mf}\;\mathrm{after}\;\mathrm{treatment}}$$

The Pearson’s Chi square test was used to test for significant differences in percentage reductions of mf between groups. The Wilcoxon signed rank test was used to test for any significant differences in mf counts, haematological and biochemical parameters before and after treatment.

All clinical manifestations observed were described based on scores defined as: 0 = normal/absent/no alteration, 5 = mild, 10 = moderate, 15 = severe and 20 = unbearable. The Kruskal and Wallis test was used to test for significant differences in vital signs. Bar charts were used to represent the mean scores of clinical signs in the different treatment groups. The distribution of mf/25mm^2^ in the different organs for animals in the different treatment arms were represented using bar charts; the Kruskal and Wallis test was used to test for significant differences in the distribution of tissue mfs. A description of the different lesions observed at the gross and histological levels were done. The mf found in the different tissues of Bab 08 was considered under the category of IVM only, because it took only this drug and died 5 hours after. All tests were performed at 5% significance level.

## Results

### Reduction of peripheral mf counts after ivermectin treatment

The pre-treatment *Loa* microfilaraemia load ranged from 19,800–124,700 mf/mL; median 39,500 mf/mL of blood and the post-treatment microfilarial load ranged from 160–34,660mf/mL; median 12,900 mf/mL of blood.

**Fig 2:**
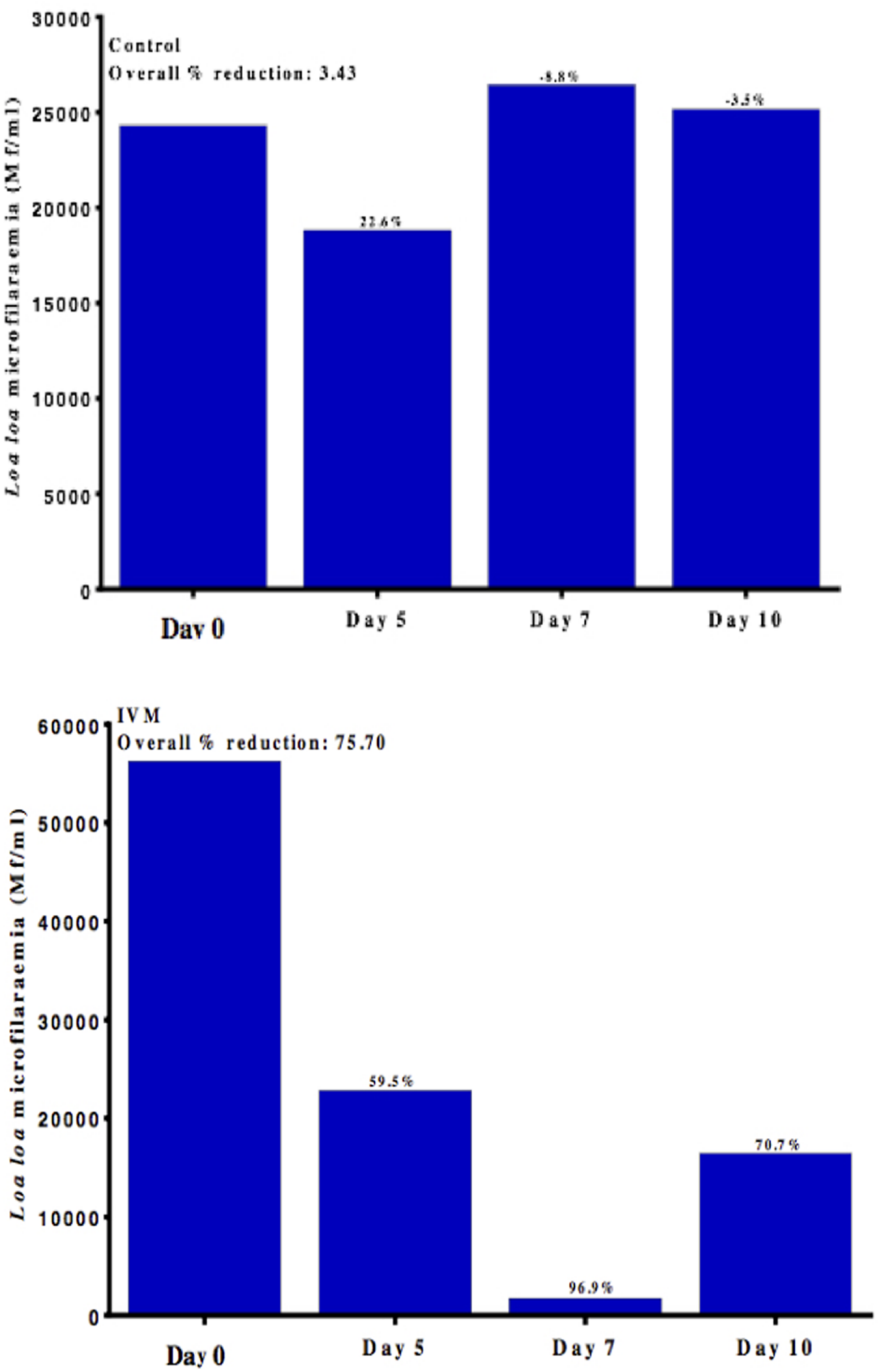
Changes in *Loa* mf loads a different time points. a. Untreated animals. b. Following ivermectin treatment.

The untreated animals showed an overall percentage fluctuation of 3.4% over the 10 days period monitored. After 5 days of monitoring, these animals showed a reduction of 22.6% whereas the animals monitored for 7 and 10 days showed increases in the mf numbers of 8.8% and 3.5%, respectively from day 0 (Fig 2A).

For all animals administered IVM (IVM alone, IVM+ASA and IVM+PSE), the overall reduction in mf load after ivermectin treatment was 75.7%. Animals monitored for 7 days recorded the highest (96.9%) reduction in mf loads following treatment whereas those monitored for 5 days recorded the lowest (59.5%) of the reductions seen in mf loads with treated animals (Fig 2). There was a significant difference (p = 0.02) in percentage reduction in animals treated at different time points. For each treated animal, there was a significant drop in microfilaraemia p<0.05, Table 1. Bab 05 (with an initial *Loa* mf load of 10,080 mf/mL) recorded the highest (98.4%) reduction in mf numbers while Bab 02 (with an initial *Loa* mf load of 79,660 mf/mL) recorded the lowest (61.8%) reduction in mf numbers after treatment with IVM. The percentage reduction in individual animals treated with IVM is shown in Table 1.

**Table 1:** Blood peripheral mf loads before and after different treatments.

Code	Drug Administered	Exp. Group	No. Days Monitored	Mf load BF	Mf load AF	Percentage Reduction (%)	Z (Wilcoxon Signed Rank Test)	P-value
Bab04	None	3	10	21580	25160	-16.4	---	---
Bab10	None	1	5	19800	18820	4.9	---	---
Bab11	None	2	7	31560	26460	16.2	---	---
Bab05	IVM	2	7	10080	160	98.4	-5.385	0.002
Bab07	IVM	1	5	92780	10940	88.2	-3.464	0.001
Bab12	IVM	3	10	35760	6000	83.2	-4.796	0.004
Bab08*	IVM	1	-----	124700	----	-----	-----	-----
Bab01	IVM+ ASA	3	10	36400	12900	64.6	-6.164	0.001
Bab06	IVM+ ASA	2	7	42600	1860	95.6	-5.292	0.002
Bab09	IVM+ ASA	1	5	101140	34660	65.7	-3.742	0.003
Bab02	IVM + PSE	3	10	79660	30460	61.8	-6.164	0.002
Bab03	IVM + PSE	2	7	51480	3220	93.7	-5.385	0.003

*Animal died 5 hours after ivermectin treatment; BF = before treatment, AF = after treatment

**Fig 3:**
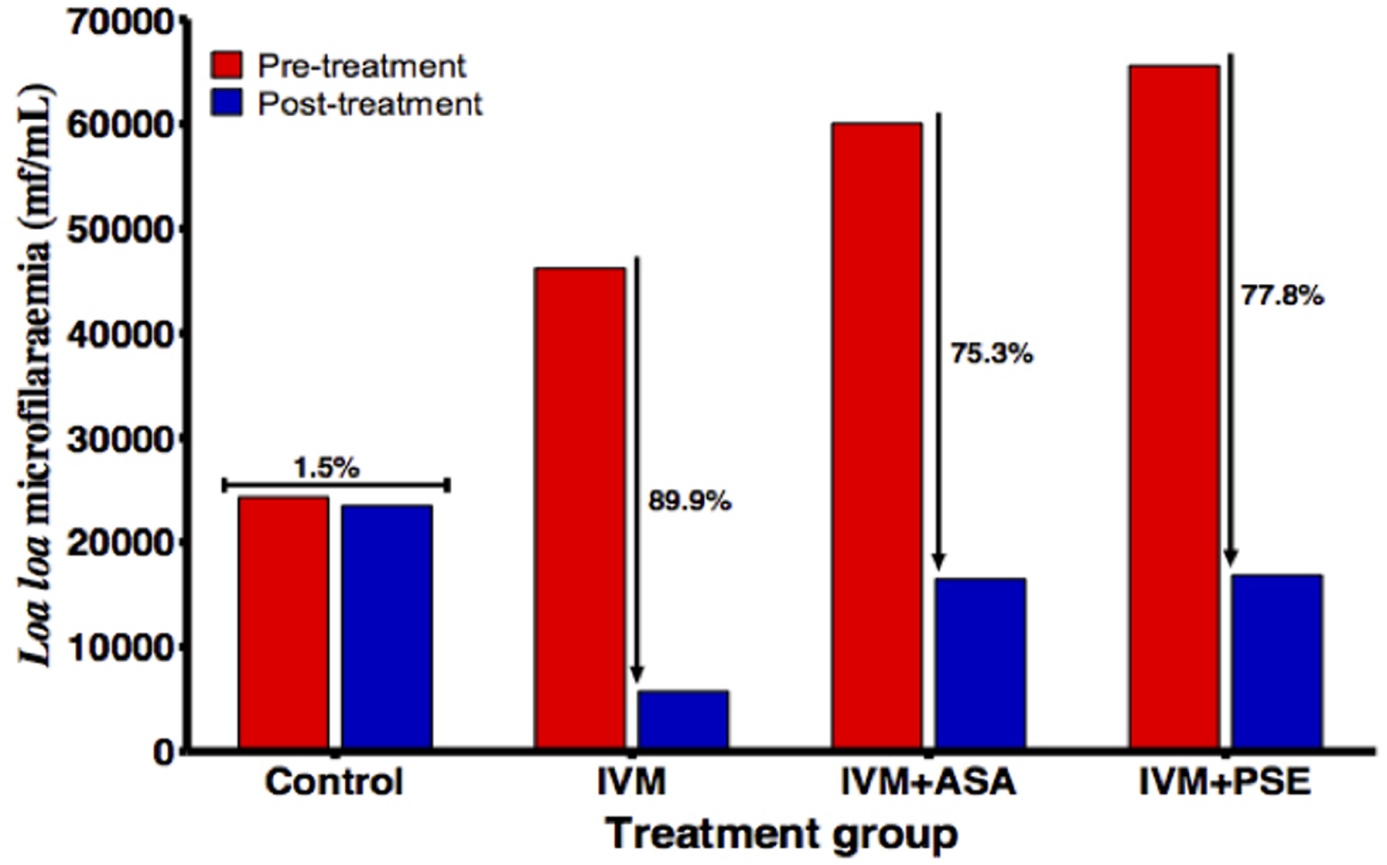
Changes in *Loa* mf loads with different treatment regimes. : ivermectin alone (IVM), ivermectin and aspirin (IVM+ASA), and ivermectin and prednisone (IVM+PSE).

With respect to treatment groups, the highest percentage reduction (89.9%) in peripheral mf loads was recorded in animals that were administered IVM alone (Fig 3). There was a significant difference (p = 0.03) in percentage reduction in animals in the different treatment arms administered ivermectin.

### Changes in haematological parameters following treatment

Amongst all the haematological parameters monitored there was a significant difference between the pre- and post-haematological parameters when looked globally (p = 0.028). Haemoglobin values before treatment ranged from 12–16 g/ dL; median 14.7 g/dL while the post treatment values ranged from 12–16 g/dL; median 12.8 g/dL. However, the Hb values pre- and post-treatment remained the same (12g/dL) in animals which received IVM + PSE while for animals that received IVM alone and IVM + ASA reduced post treatment by 3 g/dL and 1 g/dL, respectively.

However, in untreated animals increases were noticed in the RBC numbers and decreases noticed in the WBC, neutrophil, mononuclear numbers before and after treatment, significant differences were not noticed in the values for these parameters pre and post treatment between the different treatment groups, (p>0.05) (S1 Table).

Although significant differences were not recorded (p>0.05) between the different treatment groups, IVM administered alone caused increases in the values pre and post treatment for WBC, eosinophils and mononuclear lymphocytes while a decrease in values pre and post treatment was noticed in neutrophil numbers (S1 Table).

IVM +ASA co-administration caused increases in the values pre and post treatment for neutrophils and decreases in RBC, WBC, eosinophils, mononuclear lymphocytes, although no significant differences (p>0.05) were recorded in these values between the different treatment groups (S1 Table).

IVM + PSE co-administration caused increases in the values pre and post treatment for RBC and decreases in WBC, neutrophils eosinophils and mononuclear lymphocytes, although no significant differences (p>0.05) were recorded in these values between the different treatment groups (S1 Table).

### Changes in biochemical parameters following treatment

The different biochemical parameters monitored did not show any significant changes in values pre and post treatment (p>0.05). The changes in all biochemical parameters before and after treatment are shown in S2 Table.

In untreated animals though increases were noticed in the SGPT, SGOT, γ-GT, creatinine and glucose levels numbers while decreases were noticed in the calcium and potassium numbers before and after treatment, significant differences were not noticed in the values for these parameters pre and post treatment, between the different treatment groups (p>0.05) (S2 Table).

Although significant differences were not recorded (p>0.05) between the different treatment groups, IVM administered alone caused increases in the values pre and post treatment for SGOT, glucose and calcium, while decreases in values pre and post treatment were noticed in SGPT, γ-GT, creatinine and potassium (S2 Table).

IVM +ASA co-administration caused increases in the values pre and post treatment for γ-GT, no changes in calcium levels and decreases in SGPT, SGOT, creatinine, glucose and potassium although no significant differences (p>0.05) were recorded in these values between the different treatment groups (S2 Table).

IVM + PSE co-administration caused increases in SGPT, SGOT, γ-GT and glucose levels and decreases in creatinine, calcium and potassium levels although no significant differences (p>0.05) were recorded in these values between the different treatment groups (S2 Table).

### Clinical manifestations observed in baboons

**Fig 4:**
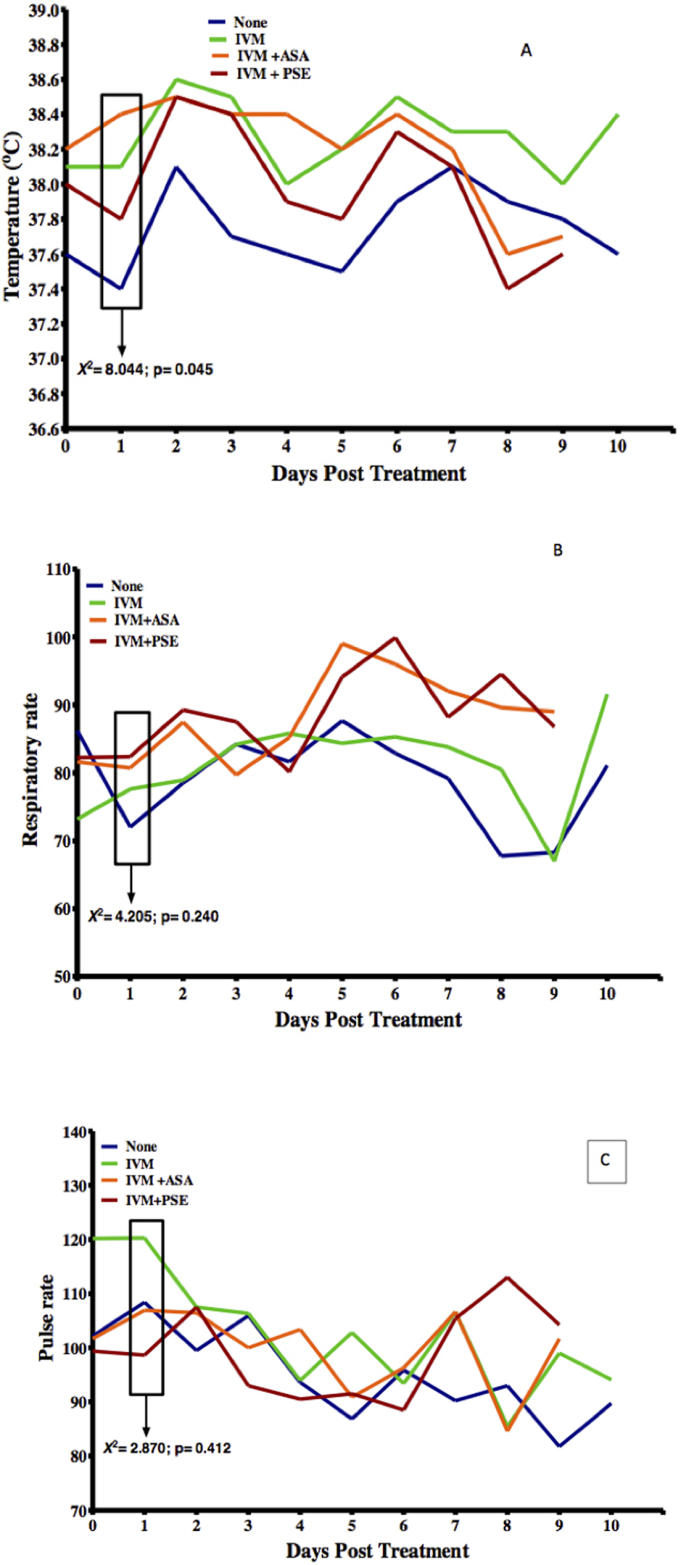
Variations in clinical presentations after ivermectin treatment (A = temperature; B = respiratory rate; C = pulse rate).

**Fig 5:**
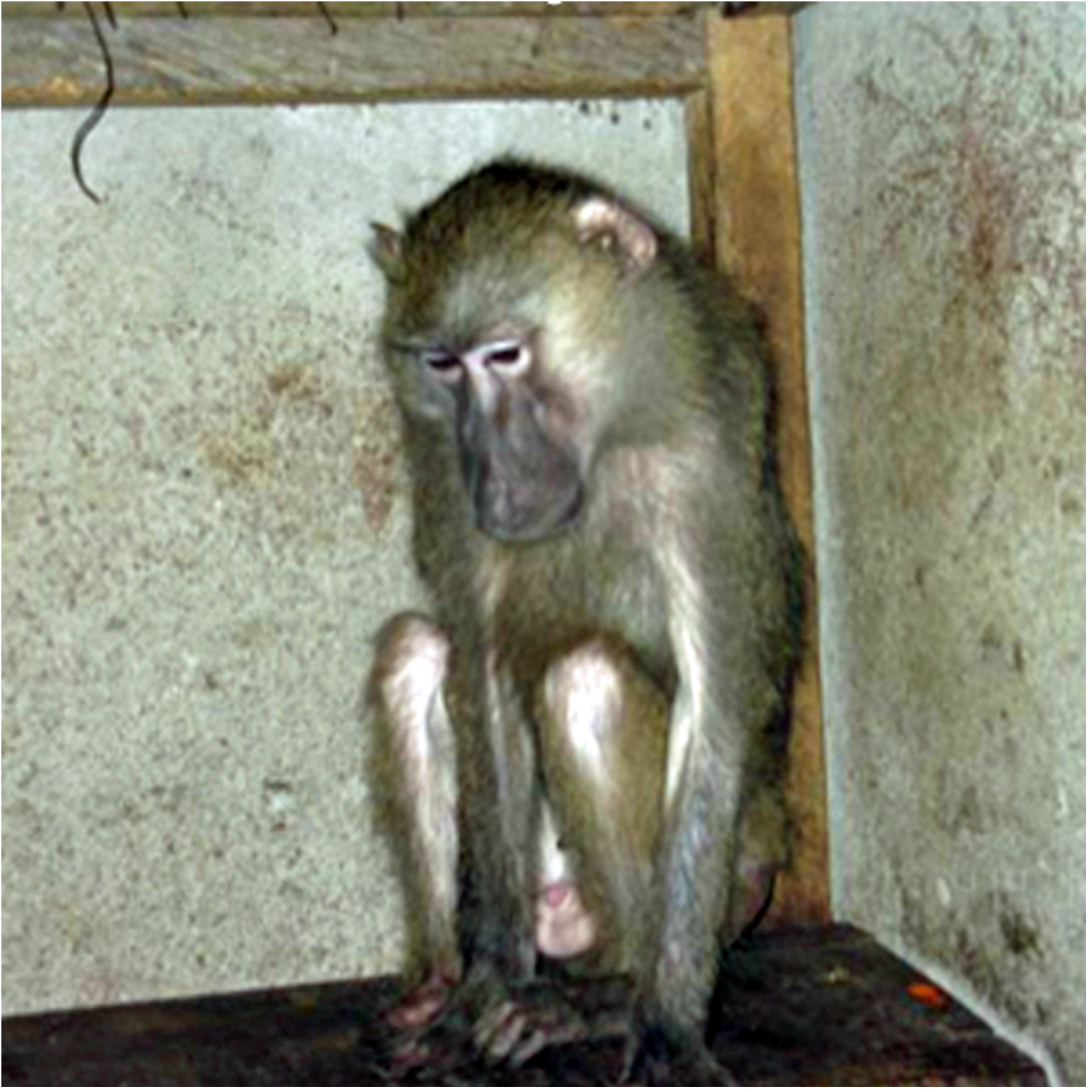
The typical behavioral response after treatment: Depression and reluctance to participate in normal activities.

In all animals administered IVM at a standard dose of 150μg/kg on Day 0, they did not show any clinical manifestations, however, Bab 08 (with an initial *Loa* mf load of 124,700mf/mL) became unbearably restless, lost appetite and died 5 hours after treatment. On the second day of observation post treatment, the following clinical manifestations being observed: an increase in the observed temperature and respiratory and pulse rates. The temperature increased to between 35–40.4°C with a mean of 38.18±0.7752°C (normal range: 37.56–39.17°C) and respiratory rates increased to 49-117/min with a mean of 85.25±13.91 breaths per minute (normal range: 30–70 breaths per minute). The pulse rates ranged from 58–141 beats per minute with a mean of 98.78±18.64 beats per minute (normal range: 120–180 beats per minute). A significant difference (p = 0.045) was recorded in body temperature day 1 post treatment in the different treatment groups while no significant differences (p>0.05) were recorded in the other vital signs between animals in the different treatment groups, (Fig 4A–4C). The animals became withdrawn 48 hours after IVM treatment (Fig 5).

**Fig 6:**
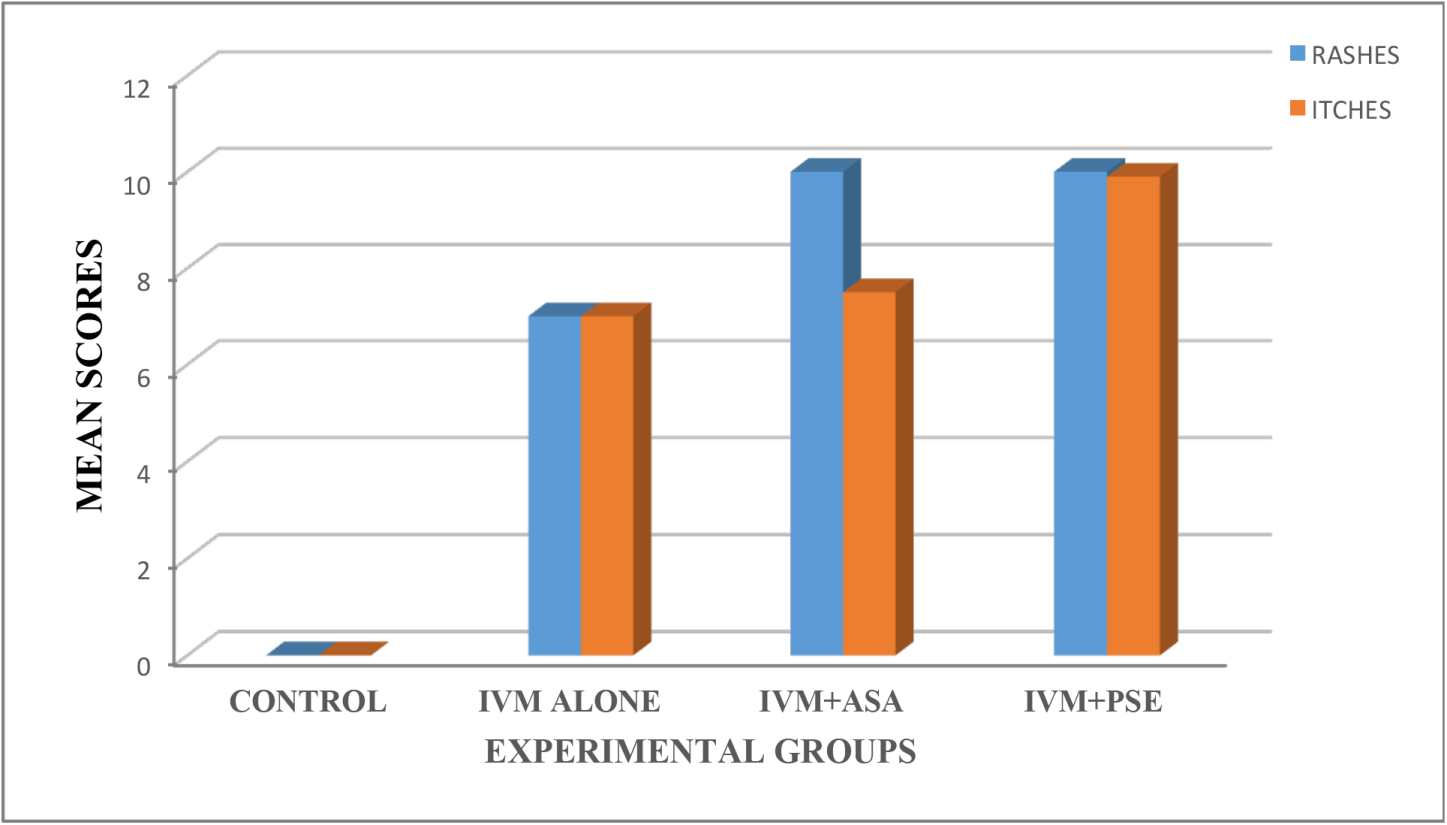
Dermatological responses in baboons after treatment. Score for rashes and pruritus in baboons following ivermectin treatment (Ranges used = 0: Normal/Absent/no alteration, 1–5: Mild, 6–10: Moderate, 11–15: Severe and 16–20: Unbearable).

Other clinical manifestations observed on this day were body rashes and itches (Fig 6A), gland pain and gland tenderness, pinkish ears, swollen face, conjunctival haemorrhages, loss of appetite, muscle ache, slight discharge form the eyes and nostrils and diarrhea. The mean scores for rashes and itching observed in the various groups are shown in Fig 6B. Untreated animals did not show any clinical alterations. On the third day after the administration of ASA (1 tablet of 500mg 2 times a day for two days), the observed rashes and itches became absent or mild in the animals in this treatment arm whereas after the administration of PSE (20mg 2 times a day for two days), the observed rashes and itches remained moderate.

### Gross pathological findings

**Fig 7:**
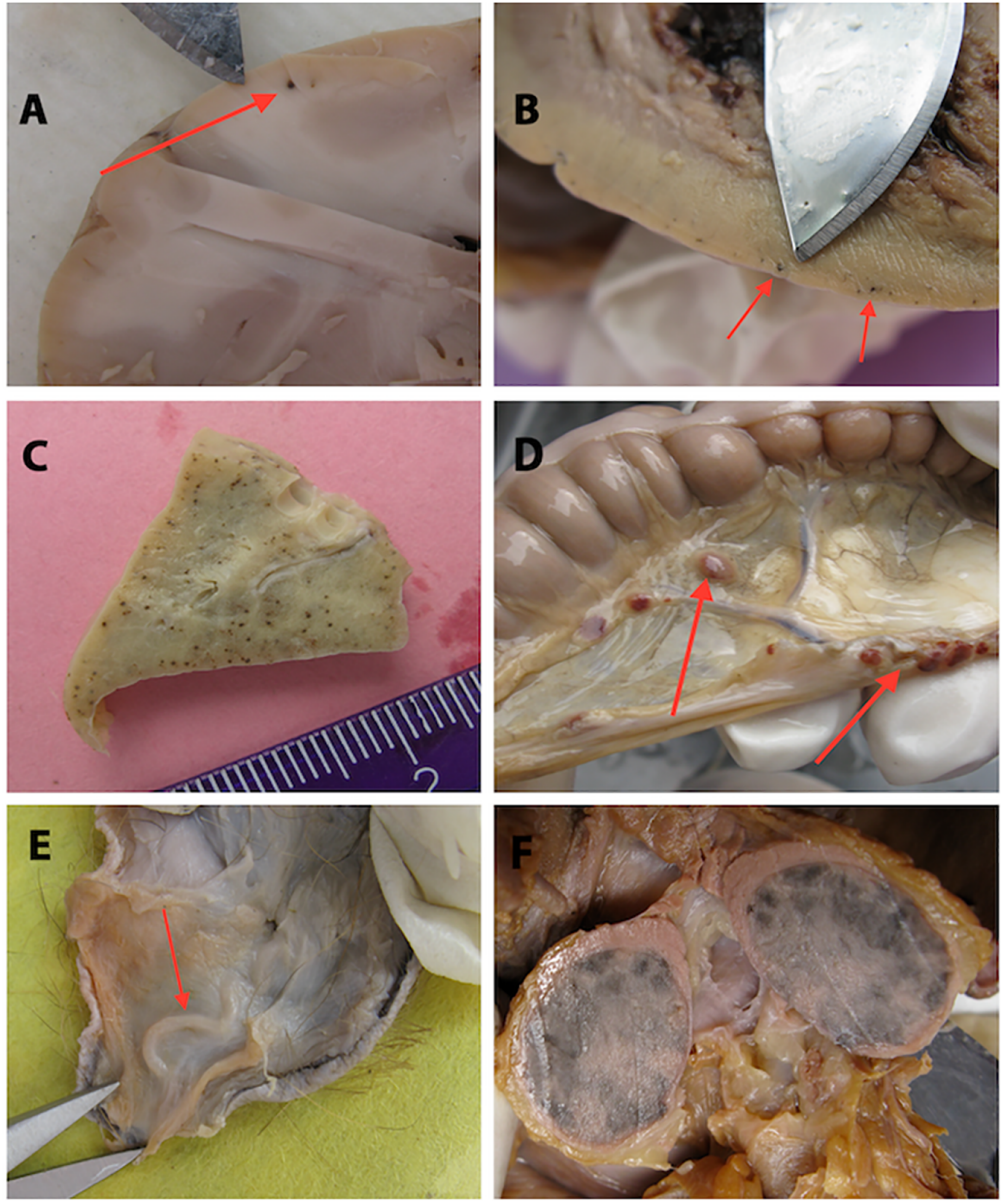
Macroscopic changes following ivermectin treatment. A. Petechial haemorrhages in the central nervous system, B. Cardiac muscle section showing petechial haemorrhage, C. Petechial haemorrhages in the lungs, D. Haemorrhagic lymph nodes in the omentum, E. Adult worm lesion present in the deep subcutaneous tissues, and F. Newly formed splenic tissues approximately 18 months after splenectomy.

The macroscopic changes seen included changes that were primarily those of a vasculo-pathologic nature (e.g. haemorrhages, hyperaemia/injection, pleural effusions, etc.). Petechial haemorrhages were seen in the CNS, the lungs, the conjunctiva, the cardiac tissues, the peritoneum and the omentum. Adult worms were present in the deep subcutaneous tissues as well as the development of pleural effusions and enlarged and haemorrhagic lymph nodes, the skin showed excoriations and pachydermia on the limbs. These changes were seen in all groups given ivermectin, occurring at various levels, except in the untreated group (which showed none of these changes). The majority (11/12) of baboons, all whom had been splenectomised as part of inducing the infection with *L*. *loa*, were seen to have regrown “new splenic tissue”. Most of these new tissues were variable in shape, located in the supra-renal area on the left or at least within 10 centimeters of this area; in two animals there were two independent such new organs in this anatomical location. These new tissues were most usually round ball-like and on sectioning were dark red in colour with a distinct fibrous capsule with some visible trabeculae. The different pathological changes seen are shown in Fig 7A–7F.

### Distribution of tissue microfilariae

Microfilariae were visible histologically in all animals. The brain and kidneys carried high numbers of mf in all treatment groups whereas lower numbers of mf were present in organs such as the diaphragm, pancreas and intestine in all other treatment groups except in the untreated infected group which recorded no mf in the diaphragm and pancreas. No significant differences (p>0.05) were detected in the distribution of mf within the different organ tissues between any of the treatment groups including the kidneys (p = 0.794), except in the brain tissues, which had a significant more mf (p = 0.04) in animals from different treatment groups.

**Fig 8:**
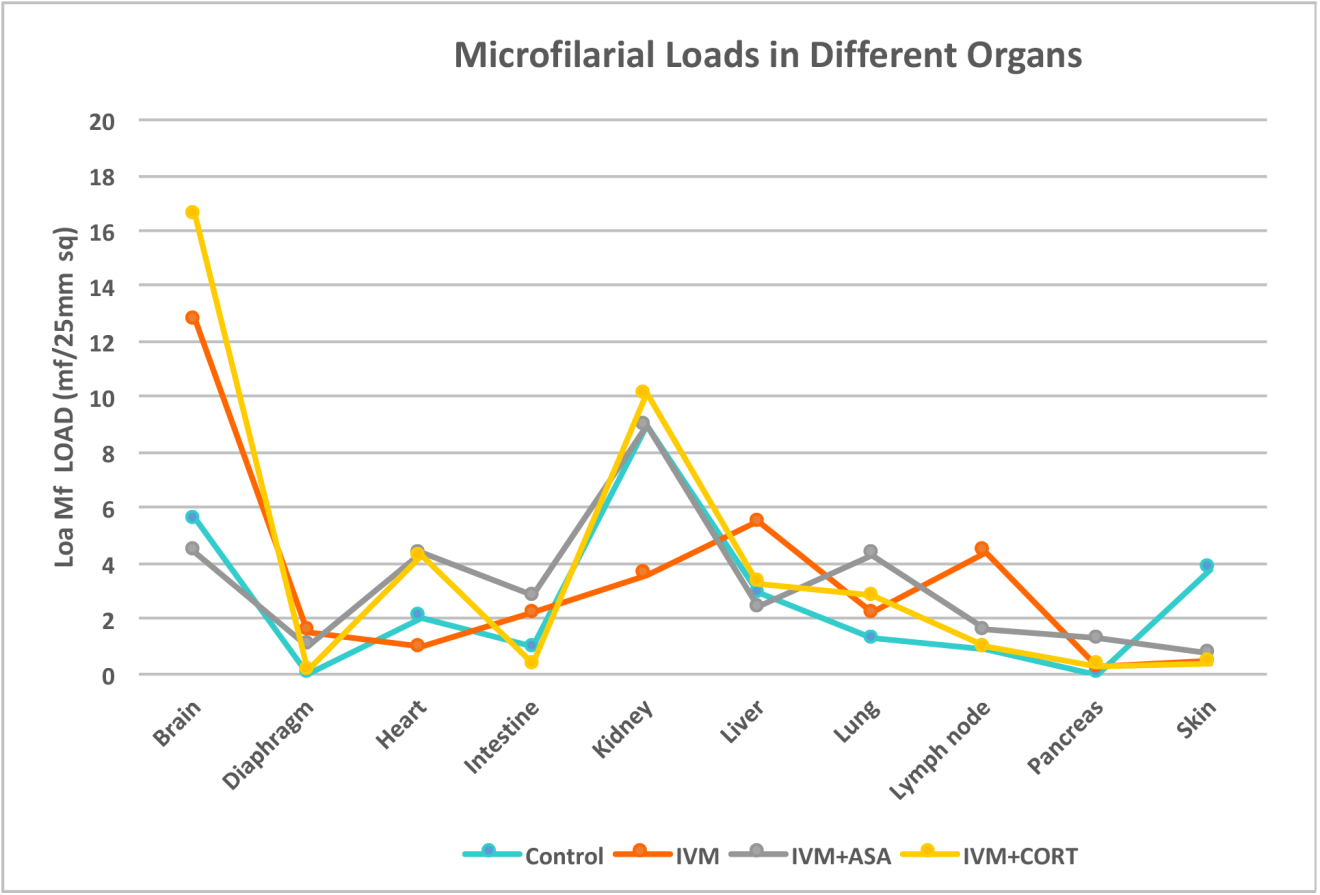
Distribution of L. loa mf in the tissues of different organs in the four experimental groups: Control animals, animals given ivermectin alone, animals given ivermectin and aspirin animals, and animals given ivermectin and prednisone.

In untreated animals, mf was found in the tissues of all organs observed except in the diaphragm and pancreas. The highest numbers of mf counted in all observed tissues were in the brain (4.2 mf/25 mm^2^ and kidneys (5.4 mf/25 mm^2^) of these animals. The distribution of mf in the tissues of animals that served as controls can be seen in Fig 8. In animals treated with IVM alone, mf were seen in all the observed tissues. In these animals, the mf increased greatly in most of the tissues. In the brain the increase to 13.8 mf/25 mm^2^ was 3 times; in the kidneys the decrease to 2 mf/25 mm^2^ in animals administered IVM alone was 3 times lower than that of animals infected but remained untreated and in the lymph nodes the increase to 4 mf/25 mm^2^ was 4 times-that of control animals. The distribution of mf in tissues from the different organs of animals that received IVM, can be seen in Fig 8.

In animals administered aspirin after ivermectin treatment, mf were also seen in the observed tissues. In these animals it was noticed that the mf seen in the tissues decreased greatly after ASA was administered. In the brain the decrease to 2.8 mf/25 mm^2^) was 5 times; kidneys the decrease to 8 mf/25 mm^2^) was 2 times; in the liver the decrease to 2.8 mf/25 mm^2^) was 2 times; in the lymph node the decrease to 1.8 mf/25 mm^2^ was 3 times -that of animals administered IVM alone. The distribution of mf in tissues from the different organs of animals that received IVM + ASA, can be seen in Fig 8.

**Fig 9:**
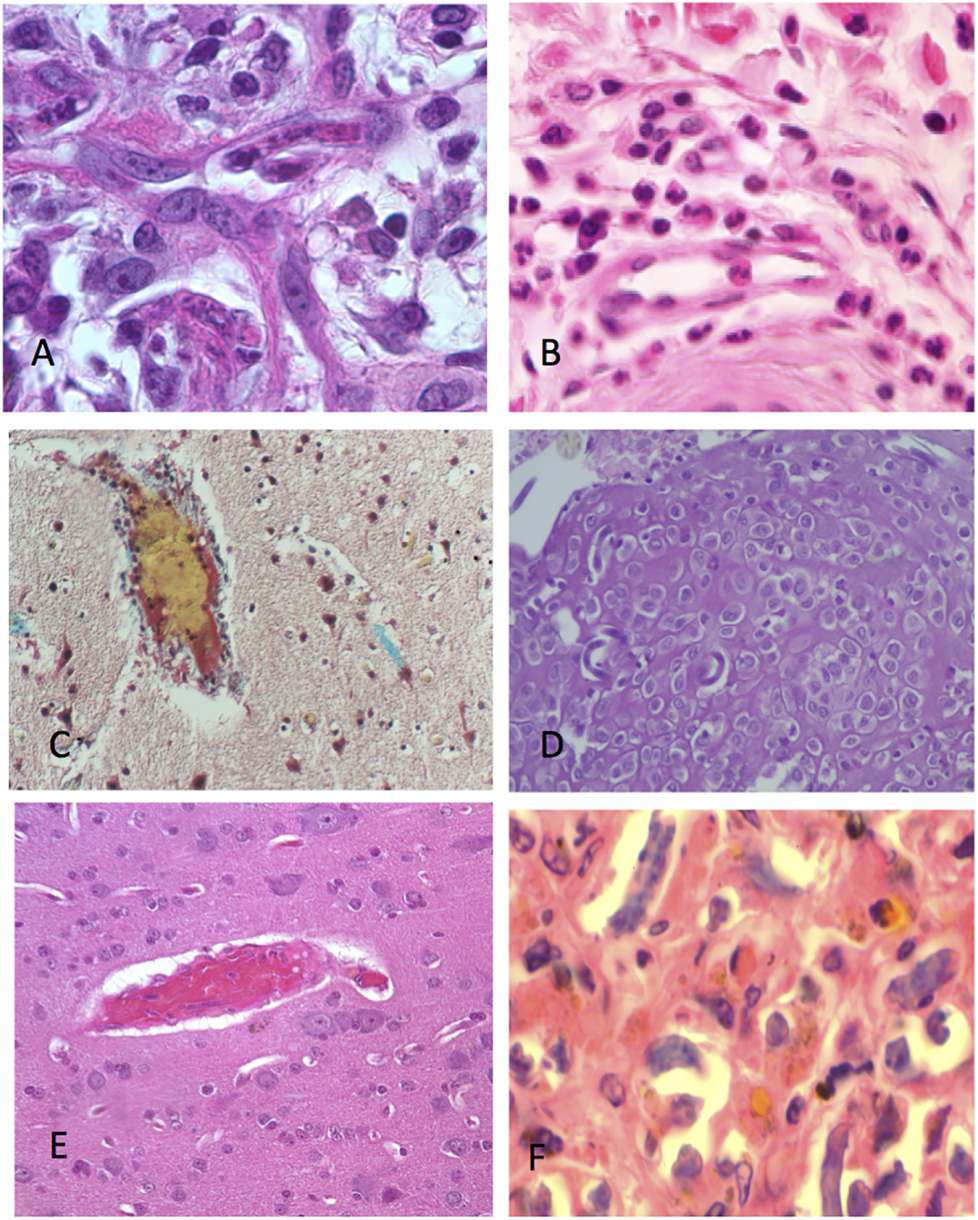
Microscopic lesions present in the untreated and in the first 72 hours after treatment. A. Microfilariae in dermal vessels. B. Eosinophil accumulation in the tissues, a typical response in the first 24 hours, C. Blood vessel free of mf- many normal vessels can also be seen free of mf in this phase, D. Accumulation of mf in the lymphoid tissues, an event that is common within the first 2–3 days, E. Fibrin deposition (red) on the walls of a cerebral vessel. F. Area of mf degeneration and eosinophil accumulation/degranulation in a lymph node.

Microfilariae were equally observed in the tissues of animals administered prednisone after IVM treatment. Here it was noticed that mf numbers increased greatly in the brain tissues. In the brain the increase in mf to 17.4 mf/25 mm^2^ was 1.3 times that of animals administered IVM alone. In the kidneys, liver and lungs decreases to 10 mf/25 mm^2^, 3.8 mf/25 mm^2^ and 2.8 mf/25 mm^2^, respectively, were noticed which were 0.9, 1.5 and 0.6 times, respectively as compared to those of animals which were administered IVM alone. The distribution of mf in tissues from the different organs of animals that received IVM, can be seen in Fig 9B.

### Lesions in organs of baboons following treatment with ivermectin and other drugs

The major cellular changes seen, and which were present in all the ivermectin treated animals, were:

1. The presence of microfilariae of varying degrees of degeneration in small vessels, these parasites were frequently associated with either fibrin deposition, endothelial alteration (cellular swelling) including damage to the integrity of the blood vessel and the presence of extravascular erythrocytes (haemorrhage).
2. The increased presence of eosinophil leucocytes and other chronic inflammatory types in certain tissues and organs, sometimes in large numbers. c. Local evidence of microfilarial death, i.e. degenerating microfilariae.

**Fig 10:**
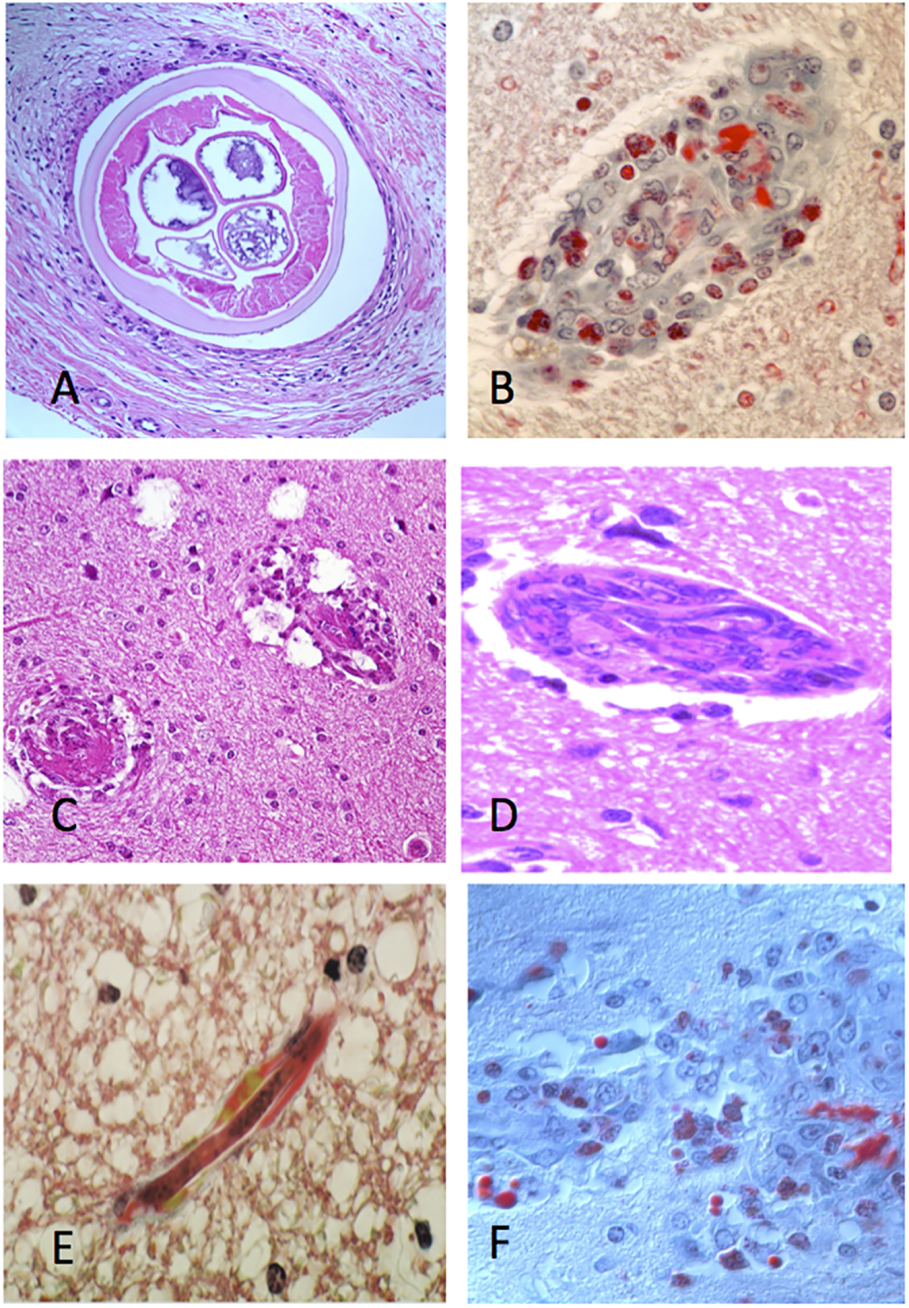
Microscopic lesions present in the treated animals more than 72 hours after treatment. A. Adult L. loa worm in connective tissue beneath the skin. B. Blocked CNS vessel comprised of eosinophils, fibrin, macrophages and parasite debris. C. Blocked CNS vessels with associated damage (vacuolation of the parenchyma). D. Intact microfilariae caught in a cellular intravascular mass in the CNS, E. A degenerating mf in a capillary of the CNS and surrounded by fibrin. F. Area of vascular and parenchymal damage in the CNS predominately filled with macrophages and eosinophils.

The lesions found in untreated animals and in the first 72 hours after treatment with ivermectin included eosinophilic accumulation in the tissues, accumulation of mf in the lymphoid tissues, fibrin deposition on the walls of cerebral blood vessels and mf degeneration and eosinophilic accumulation/degranulation in a lymph node. These changes can be seen in Fig 10A–10F.

After 72 hr. following treatment, the observed lesions included blocked CNS vessels comprised of eosinophils, fibrin, macrophages and parasite debris, blocked CNS vessels with associated damage (vacuolation of the parenchyma), intact microfilariae caught in a cellular intravascular mass in the CNS, degenerating mf in blood capillaries of the CNS and surrounded by fibrin, and an area of vascular and parenchymal damage in the CNS predominately filled with macrophages and eosinophils. The different lesions seen more than 72 hours post treatment with ivermectin can be seen in Fig 10A–10F. The presence and extent of histo-pathological changes seen in the different organs of the various treatment groups can be seen in Table 2. The distribution of different lesions seen in the different tissues indicate that they were mild in untreated animals while in animals administered ivermectin alone, these lesions were moderate; the distribution of these lesions between treatment groups can be seen in Table 3.

**Table 2:** Presence and extent of histo-pathological changes in different organs (No. of animals per group).

Organ	Infected untreated (1)	Infected + ivermectin (3)	Infected + ivermectin + aspirin (3)	Infected + ivermectin + prednisone (3)
CNS	**-**	**+**	**+**	**+**
Lungs	**-**	**+**	**+**	**+**
Lymph nodes	**-**	**++**	**+**	**-**
Liver	**+**	**+**	**+**	**+**
Kidney	**+**	**++**	**++**	**++**
Skin	**+**	**+++**	**+++**	
Spleen*	**++**	**++**	**++**	**++**
Heart	**-**	**++**	**+**	**+**
Peritoneal cavity	**-**	**+**	**-**	**_**
Thoracic cavity	**-**	**+++**	**+**	**++**
Ocular tissues	**-**	**+**	**_**	**_**
Intestine	**-**	**+**	**+**	**+**

* These are newly growing splenic structure.

Score: + = mild; ++ = moderate; +++ = severe

**Table 3:** Distribution of lesions between the different treatment groups (No. animals per group).

Pathological change	Infected untreated (1)	Infected + ivermectin (3)	Infected + ivermectin + aspirin (3)	Infected + ivermectin + prednisone (3)
Intravascular lesions with mf	-	++	+	++
Microfilarial destruction	+	++	++	++
Micro-haemorrhages	-	+	+	+
Macro-haemorrhages	-	+	+	+
Eosinophil infiltration to lymph nodes	-	++	(+)	(+)
Eosinophil infiltration to other tissues	-	++	+	++
Macrophage responses to microfilariae	+	++	+	+
Macrophage responses to adult worms	+	++	++	++

Score: + = mild; ++ = moderate; +++ = severe

## Discussion

In this report the clinical spectrum and pathology related to the treatment of hypermicrofilaraemic *Loa* infected baboons with ivermectin is presented for the first time. After treatment with ivermectin, microfilaraemia decreased significantly in all treated animals with up to 98.4% reduction in one animal (Bab 05). This finding confirms the findings of earlier authors who had shown ivermectin to have a marked microfilaricidal effect on *L*. *Loa*. A study by Kamgno *et al*. [23] demonstrated that one month after a single dose with ivermectin, microfilarial loads fall to <20% of their initial values and this suppression of microfilaraemia can persist for at least a year [24]. The reduction of microfilariae numbers in peripheral blood could probably be due to the fact that ivermectin treatment induces the *Loa* microfilariae to flee from the blood circulation into other body fluids such as the pleural and peritoneal fluids as a result of vascular breaking or due to the death of these mfs.

The drastic killing of circulatory mf could lead to the blockage of blood vessels in the different tissues and organs. When this happens, an inflammatory reaction sets in activating the fibrin pathway whereby fibrin clots are deposited in the tissues. The release of inflammatory cytokines such as interleukin (IL)-1, IL-6, IL-17 and tumor necrosis factor alpha (TNF-α) which have been incriminated as being part of pathological signal cascades in brain diseases [25] could have been released in these animals, although these cytokines were not measured. The formation of fibrin clots could result in the blockage of blood vessels in the different tissues and organs causing tissue anoxia and finally death.

It is not clear as to the significant changes between the pre and post treatment haemoglobin levels. However, no animals were found to be clinically anaemic, and as the animals were fed on a rich protein-diet it is likely that this variation in parameter was not a major characteristic of ivermectin’s effect on haemoglobin. In all treated animals, there was an increase in the total white blood cell counts, eosinophils, and neutrophils. The increase in WBC counts is similar to what Ducorps *et al*. found out in their study [10]. This increase is probably due to an enhancement in the production of many more leukocytes to reduce the severity of Mazzotti reaction and to fight the infection in general. The increase in eosinophils in this study is similar to what Martin-Prevel *et al*. [26] noticed where treatment with ivermectin causes an increase in eosinophils. The global changes in eosinophil counts recorded after treatment with ivermectin in patients harbouring *L*. *loa* is similar to those observed in onchocerciasis patients after treatment with ivermectin or DEC [27, 28]. This could probably be due to the preponderant role of eosinophils in the development of the Mazzotti reaction whose severity is associated with eosinophil sequestration and activation-degranulation [29, 30]. The increase in neutrophils observed is probably because these cells are the hallmarks of acute inflammation given the fact that the migration and degranulation of eosinophil in the skin releases parasite specific antigens during cell death that induces a pro-inflammatory response. The general increase observed in SGPT, SGOT and γ-GT levels after ivermectin treatment could probably be due to hepatocellular damage or obstruction in bile flow cholestasis [31, 32]. Generally, this drug has not been associated with acute or chronic liver injury, although Veit *et al*., [33] realised that treatment with a single dose of ivermectin resulted in mild hepatotoxicity. Elevations in SGPT and SGOT in blood are increased in conditions in which cells are damaged or dead. It is also important to note that the elevations in serum liver enzymes can also be secondary to enzyme induction without hepatic pathology [34]. The elevations of creatinine post-treatment in control animals was not surprising because during the course of infection after inoculation of mfs into these animals, its’ levels were already high. This could probably be related to kidney dysfunction [35] as a malfunction in glomerular filtration results in the retention of substances including urea and creatinine. Generally, creatinine levels dropped in all animals that took ivermectin alone, or ivermectin together with other treatments. This decrease is probably due to the fact that the kidneys were more efficient in excreting the compound. The elevated serum glucose level observed may have resulted from increased mobilization of glucose for metabolism or may be due to reduced glucose uptake into cells caused by ivermectin [36] or a possible modulation of the capacity of the renal tubule by the drug to reabsorb glucose actively from the blood [36]. The decrease in potassium levels in untreated animals could probably be due to a reduced intake in their diet [37] or a decreased sensitivity of the nephron to aldosterone and other mineralocorticoids responsible for reabsorption and retention of electrolytes, respectively. However, the drop in potassium levels in animals administered ivermectin and prednisone is probably due to the fact that prednisone is known to cause more urination as more blood moves to the kidneys. It is probable that as more urine is sent out, more potassium ions are also lost along with it from the body in these animals or reduced intake in their diet [37]. The changes in calcium ions pre and post treatment could probably be related to variations in diet intake of this mineral [38].

Generally, all animals administered ivermectin became withdrawn 48 hours following treatment. This observation is in line with the results of earlier studies [12, 13] which revealed that symptoms appeared within two days of ivermectin treatment in patients who were previously healthy. This withdrawal attitude could be due to stress that developed due to loss of coordination, reduced alertness that can progress to stupor or coma. A withdrawn attitude is a neurological manifestation that is related to blockage and reduced blood flow supplying nutrients and oxygen to the cells that could be due to the destruction or death of mf in the brain and other tissues. A possible scenario for the pathogenesis of the animal that died 5 hours after ivermectin treatment could be that at the time of ivermectin treatment, this animal harbored a *Loa* mf load (124,700mf/mL) greatly exceeding the threshold associated with the risk of post-ivermectin Loa encephalopathy. The initial *L*. *loa* microfilarial load has been demonstrated to be the main risk factor for the development of serious reactions and an association has been found to exist between *L*. *loa* load and the occurrence of marked or serious reactions has been found to be significant above 8,000mf/mL with the association being close and stable [12,13].

In all animals treated with ivermectin, there was the development of body rashes and itches in otherwise healthy animals just one day after treatment. The development of rashes could probably be due to an enhancement in the production of many more leukocytes (such as eosinophils and neutrophils) to reduce the severity of Mazzotti reaction and to fight the infection in general; these cells being the hallmarks of acute inflammation. The presence of haemorrhages with evidence of *L*. *loa* mf seen in the brain, eyes and other organs of treated animals could probably be due to the fact that ivermectin results in rapid killing of Loa mf in these tissues. The obstruction of blood capillaries by these dead worms in these organs could lead to an ischemic reaction, increased pressure within the capillaries, rupture of the affected vessel(s) and haemorrhagic suffusion. This observed vasculopathy with evidence of *L*. *loa* as a likely etiology in the brain tissue therefore confirms the definite case of *Loa* encephalopathy as put forth by the group of independent experts who consulted for MDP immediately after the first cases of SAEs were noticed in the 1990s. Lymphadenopathy was present in all baboons that received ivermectin. Enlarged lymph nodes generally result from the accumulation of leucocytes within the lymph glands. Ivermectin, has been shown to cause an increase in the total white blood cell counts, number of eosinophils, and neutrophils [10].

**Fig 11:**
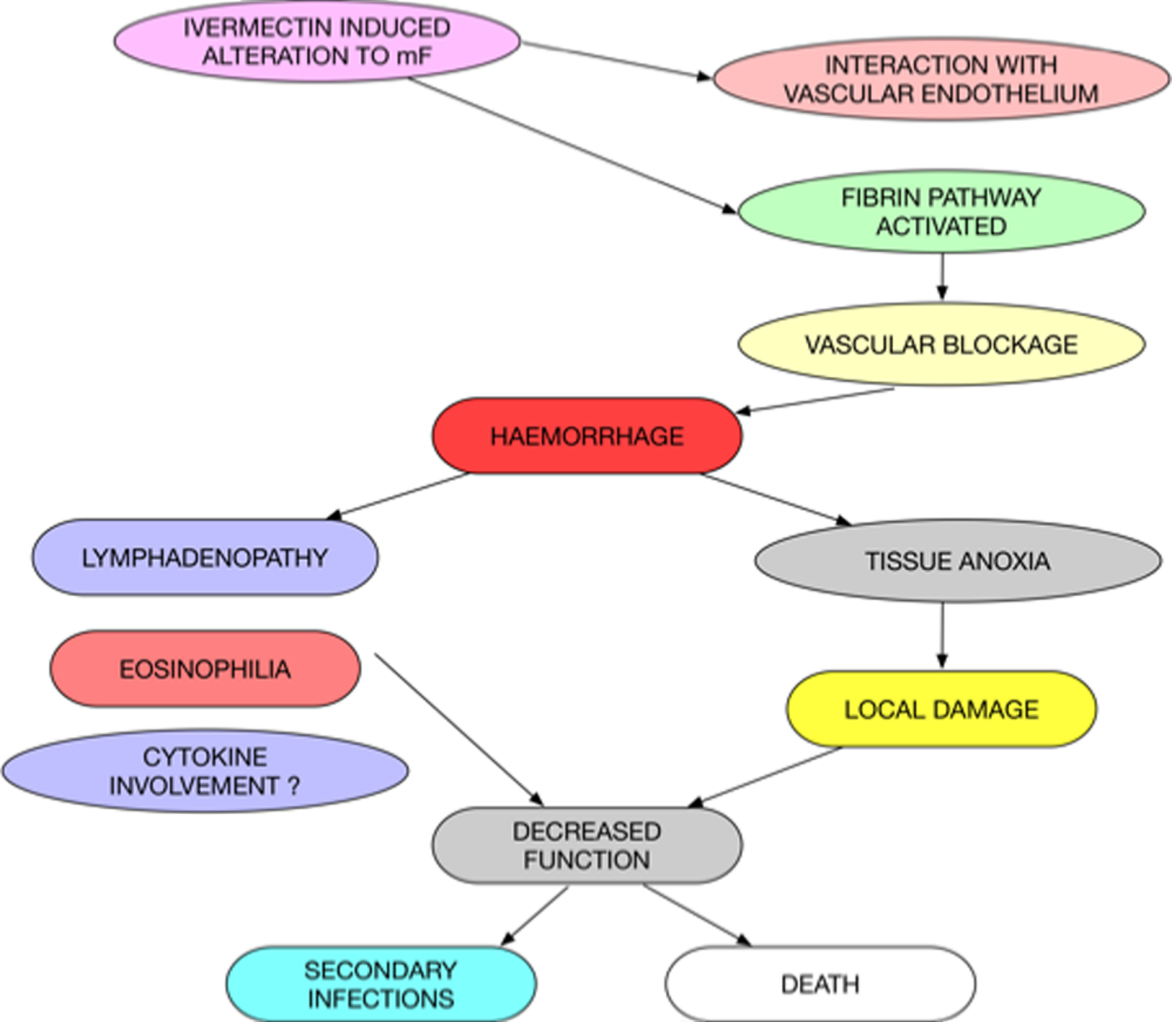
Potential pathogenesis of Loa encephalopathy following the ivermectin treatment of Loa hyper-microfilaraemic individuals.

Our results suggest that the pathogenesis of *Loa* encephalopathy is as a result of the death of mf in the blood vessels in the brain. This event causing inflammation to set in with the activation of the fibrin pathway with fibrin clots being deposited and these blocking the blood vessels in the brain and other tissues. This deposition of fibrin clots damages the walls of these blood vessels as a result of the release of inflammatory cytokines and these overall pathologic processes affecting the local tissues through tissue anoxia and other detrimental changes that eventually result in the permanent damage and in some cases the death of the host (Fig 11).

For the first time, this study has provided information on the tissues and body fluids where mf could be found after ivermectin treatment of *Loa* heavily infected individuals. Generally, organs that normally receive large amounts of blood were noticed to be harbouring many more microfilariae than those less irrigated. Given that *Loa loa* is a blood dwelling filarial worm, its presence in these highly irrigated organs is to be expected. It is not known as to why aspirin and prednisone lead to a decrease or an increase in the number of tissue sections. The presence of microfilariae in other body fluids (peritoneal and pericardial) collected at autopsy could probably be due to the fact ivermectin treatment induces the *Loa* microfilariae to flee from the blood circulation. This is an important finding which indicates that in those humans who suffer from SAEs, the serious events noticed could be due to breathing difficulties due to the accumulation of fluid in the lungs or complications such as ascites which might cause difficulties in compressing the diaphragm leading to breathing difficulties. The discovery of the presence of these effusions could serve as an important step in managing the SAEs clinically as these fluids could be drained and an intravenous infusion administered to these patients to balance fluid and electrolytes.

The primary lesions seen in the untreated infected animals were peri-portal accumulation of lymphocytes; as present in all of the animals. It is likely that in addition to the spleen, this organ and the peri-portal region is a common location for the degeneration of microfilariae. It is also possible that this occurs as a result of the initial removal of the spleen as part of the infection process, and the liver has become the default site for microfilarial destruction. It is clear that a major pathogenic event following ivermectin is a vasculopathy associated with the death and degeneration, with a cellular response, and all of this taking place intravascularly. Although there is on occasion clear damage and the resulting haemorrhaging of vessels, it is more likely that this vascular blockage results in acute parenchymal damage or dysfunction in the brain that results in the clinical condition.

Generally, in this study we found out that animals administered aspirin showed milder symptoms compared to those that received prednisone. Aspirin is part of a group of medications called non-steroidal anti-inflammatory drugs (NSAIDs), but differs from most other NSAIDs in the mechanism of action. It produces lipoxins, most of which are anti-inflammatory [39]. It is probable that the lipoxins produced by this drug aid in alleviating the symptoms observed in animals after treatment with ivermectin. The finding that prednisone aggravates the clinical manifestations observed corroborates the assertions made by Boussinesq *et al*. [40] that corticosteroids do not have any beneficial effect on the course of these clinical manifestations because they might lead to iatrogenic lethal complications.

The influx of significant number of eosinophils into the tissues shortly after ivermectin treatment is likely to be a significant contributor to the pathogenesis of the condition or at least the clinical presentation. Release of toxic mediators from the activation of eosinophils would probably be an important factor in the pathogenesis of this condition.

## Conclusions

The findings presented from this study have revealed that the ivermectin treatment of *Loa* hypermicrofilaraemic animals induces a drastic reduction in mf numbers with an 80% decrease observed in some animals. Hb was the only observed parameter that recorded a slight significant value pre and post treatment. Clinical manifestations observed after treatment are similar to what have been observed in humans such as rashes, itching, haemorrhagic conjunctiva, increased temperature, increased pulse and respiratory rates, diarrhoea, lymph node enlargement, pinkish ears, swollen face and restlessness. Macroscopic changes noticed were primarily those of a vasculo-pathologic nature (e.g. haemorrhages) seen in all groups given ivermectin, occurring at various levels, except in the untreated. Microscopically, the major cellular changes seen, and present in all the ivermectin treated animals, were: the presence of microfilariae of varying degrees of degeneration associated with either fibrin deposition, endothelial alteration (cellular swelling) including damage to the integrity of the blood vessel and the presence of extravascular erythrocytes (haemorrhage), the increased presence of eosinophil leucocytes and other chronic inflammatory types in certain tissues and organs, sometimes in large numbers and local evidence of microfilarial death. Highly vascularised organs like the brain, heart, lungs, and kidneys were observed to have more microfilariae in tissue sections. The number of mf seen in the brain and kidneys of animals administered IVM only, tripled that of control animals. Co-administration of IVM + PSE caused an increase in mf in the brain and kidneys while the reverse was noticed with the co-administration of IVM+ASA. The ivermectin treatment of Loa hyper-microfilaraemic baboons has revealed that the formation of fibrin clots and the presence of pleural and peritoneal effusions could probably be related to the development of the pathological conditions noticed in those *Loa* hypermicrofilaraemic humans who were administered ivermectin.

## Supporting information

S1 Table Biochemical findings in each treatment group. (PDF)

S2 Table Biochemical parameters pre and post treatment. (PDF)
