# Supplementary material for: Ivermectin treatment of Loa loa hyper-microfilaraemic baboons (Papio anubis): Assessment of microfilarial load reduction, haematological and biochemical parameters and histopathological changes following treatment
Source: PLoS Negl Trop Dis. 2017 Jul 7;11(7):e0005576. doi: 10.1371/journal.pntd.0005576 (PMC5533442; doi:10.1371/journal.pntd.0005576)
Supplement: S1 Table — (PDF) [file pntd.0005576.s001.pdf]

S1 Table : Biochemical findings in each treatment group

P

Exp. Group	1 (monitored for 5 days)								2 (monitored for 7 days)								3 (monitored for 10 days)							
Drug taken	None		IVM		IVM+ASA		IVM+PSE		None		IVM		IVM+ASA		IVM+PSE		None		IVM		IVM+ASA		IVM+PSE	
Name of animal	Bab10		Bab07		Bab09		Bab08*		Bab11		Bab05		Bab06		Bab03		Bab04		Bab12		Bab01		Bab02	
Sex	F		F		M		M		F		M		F		M		F		M		F		M	
	P	PT	P	PT	P	PT	P	PT	P	PT	P	PT	P	PT	P	PT	P	PT	P	PT	P	PT	P	PT
Haemoglobin (g/dl)	15	15	15	14	16	16	13	---	15	15	14	13	13	12	12	12	15	15	16	15	15	13	12	12
RBC x10^4 (Cells/ mm3)	365	350	340	330	410	420	335	---	360	360	360	355	340	330	320	325	370	400	400	412	365	335	325	395
WBC (Cells/mm3)	6700	7800	7000	7000	5800	10000	6600	---	7000	6600	7000	7000	6600	6000	6800	6400	9000	6000	7200	8000	8800	7000	7700	6600
Absolute Neutrophil	3350	1950	3150	2100	1160	3000	1782	---	2310	2442	2800	2100	1980	2700	2040	1920	2700	2400	2500	2400	2200	2660	2695	2508
Absolute Eosinophil	1340	1950	1050	1750	1276	3000	1518	---	1540	1320	1680	1750	1320	1500	1360	1280	2700	1200	1800	1600	2640	1540	1540	1518
Absolute mononuclear	2144	3900	2800	3150	3016	4000	3300	---	3150	2838	2520	2800	3300	1800	3400	3200	3600	2400	2880	3200	3960	2660	3465	2310

Bab08*: Animal died 5 hours after IVM and was not administered PSE or monitored for the required number of days. P= Pre-treatment; PT=Post treatment IVM=ivermectin; ASA=aspirin; PSE=prednisone

P
