# Supplementary material for: Ivermectin treatment of Loa loa hyper-microfilaraemic baboons (Papio anubis): Assessment of microfilarial load reduction, haematological and biochemical parameters and histopathological changes following treatment
Source: PLoS Negl Trop Dis. 2017 Jul 7;11(7):e0005576. doi: 10.1371/journal.pntd.0005576 (PMC5533442; doi:10.1371/journal.pntd.0005576)
Supplement: S2 Table — (PDF) [file pntd.0005576.s002.pdf]

P

S2. Table : Biochemical parameters pre and post treatment.

| Exp. Group | 1 (monitored for 5 days) | | | | | | | | 2 (monitored for 7 days) | | | | | | | | 3 (monitored for 10 days) | | | | | | | |
|---|---|---|---|---|---|---|---|---|---|---|---|---|---|---|---|---|---|---|---|---|---|---|---|---|
| **Drug taken** | **None** | | **IVM** | | **IVM+ASA** | | **IVM+PSE** | | **None** | | **IVM** | | **IVM+ASA** | | **IVM+PSE** | | **None** | | **IVM** | | **IVM+ASA** | | **IVM+PSE** | |
| | **Bab10** | | **Bab07** | | **Bab09** | | **Bab08*** | | **Bab11** | | **Bab05** | | **Bab06** | | **Bab03** | | **Bab04** | | **Bab12** | | **Bab01** | | **Bab02** | |
| **Sex** | **F** | | **F** | | **M** | | **M** | | **F** | | **M** | | **F** | | **M** | | **M** | | **M** | | **F** | | **F** | |
| | P | PT | P | PT | P | PT | P | PT | P | PT | P | PT | P | PT | P | PT | P | PT | P | PT | P | PT | P | PT |
| **SGPT (IU)** | 28 | 49 | 10 | 48 | 12 | 69 | 32 | --- | 7 | 38 | 42 | 12 | 17 | 34 | 16 | 76 | 41 | 13 | 43 | 18 | 79 | 19 | 23 | 30 |
| **SGOT (IU)** | 30 | 22 | 17 | 41 | 11 | 71 | 40 | --- | 20 | 41 | 35 | 23 | 9 | 38 | 27 | 53 | 37 | 28 | 33 | 37 | 88 | 24 | 41 | 66 |
| **Gamma GT (IU)** | 20 | 24 | 37 | 18 | 13 | 19 | 45 | --- | 16 | 23 | 46 | 23 | 25 | 35 | 22 | 28 | 6 | 42 | 44 | 47 | 8 | 34 | 18 | 31 |
| **Creatinine (mg/L)** | 7 | 13 | 12 | 24 | 8 | 21 | 3 | --- | 8 | 7 | 3 | 5 | 17 | 5 | 8 | 4 | 16 | 22 | 17 | 5 | 13 | 14 | 41 | 5 |
| **Glucose (g/L)** | 0.4 | 3.2 | 0.3 | 0.5 | 0.7 | 0.8 | 0.9 | --- | 1.1 | 0.6 | 0.9 | 0.4 | 1.3 | 0.6 | 1.6 | 1.8 | 0.7 | 1.0 | 0.6 | 2.4 | 1.6 | 0.7 | 0.5 | 0.9 |
| **Calcium (mg/L)** | 15 | 15 | 13 | 16 | 11 | 7 | 17 | --- | 14 | 7 | 7 | 21 | 9 | 8 | 8 | 7 | 17 | 16 | 21 | 7 | 9 | 11 | 27 | 10 |
| **Potassium (mmol/L)** | 0.8 | 3.1 | 3.9 | 4.3 | 0.9 | 2.6 | 2.6 | --- | 5.4 | | 5.7 | | 8.2 | | 5.2 | | 7.5 | | 6.8 | | 5.5 | | 6.1 | |

**Bab08*: Animal died 5 hours after IVM and was not administered PSE or monitored for the required number of days. P = Pre-treatment; PT=Post treatment IVM=ivermectin; ASA=aspirin; PSE=prednisone**

P
